# Thermodynamic Evolution of a Metamorphic Protein: A Theoretical-Computational Study of Human Lymphotactin

**DOI:** 10.1007/s10930-023-10123-7

**Published:** 2023-05-26

**Authors:** Laura Zanetti-Polzi, Isabella Daidone, Claudio Iacobucci, Andrea Amadei

**Affiliations:** 1 Center S3, CNR-Institute of Nanoscience, Via Campi 213/A, 100190 Modena, Italy; 2 Department of Physical and Chemical Sciences, University of L’Aquila, Via Vetoio (Coppito 1), 67010 L’Aquila, Italy; 3 Department of Chemical Science and Technology, University of Rome “Tor Vergata”, Via Della Ricerca Scientifica 1, 00185 Rome, Italy

**Keywords:** Fold-switching proteins, Thermodynamic evolution, Molecular dynamics, Essential dynamics

## Abstract

Metamorphic, or fold-switching, proteins feature different folds that are physiologically relevant. The human chemokine XCL1 (or Lymphotactin) is a metamorphic protein that features two native states, an $\alpha -\beta$ and an all$-\beta$ fold, which have similar stability at physiological condition. Here, extended molecular dynamics (MD) simulations, principal component analysis of atomic fluctuations and thermodynamic modeling based on both the configurational volume and free energy landscape, are used to obtain a detailed characterization of the conformational thermodynamics of human Lymphotactin and of one of its ancestors (as was previously obtained by genetic reconstruction). Comparison of our computational results with the available experimental data show that the MD-based thermodynamics can explain the experimentally observed variation of the conformational equilibrium between the two proteins. In particular, our computational data provide an interpretation of the thermodynamic evolution in this protein, revealing the relevance of the configurational entropy and of the shape of the free energy landscape within the essential space (i.e., the space defined by the generalized internal coordinates providing the largest, typically non-Gaussian, structural fluctuations).

**Supplementary Information:**

The online version contains supplementary material available at 10.1007/s10930-023-10123-7.

## Introduction

The relation between the 3-D structure of a protein, determined by the amino acid sequence, and its function has played a central role in structural biology for decades. Possibly, this concept has its roots in the co-development of enzymology and structural biology [1–3].

During the last two decades the scientific community began to recognize that a protein sequence, and ultimately a gene, can encode for multiple protein folds. Proteins can adopt a vast structural repertoire, from globular, single fold proteins to intrinsically disordered proteins (IDPs), which lack any fixed 3-D structure [4, 5]. Also, different chemico-physical conditions and, more in general, external perturbations, can relevantly affect the folding state of peptides and proteins [6, 7]. Interestingly, it is becoming evident that this structural variability allows certain proteins to interact with different partners absolving to multiple functions.

**Fig. 1:**
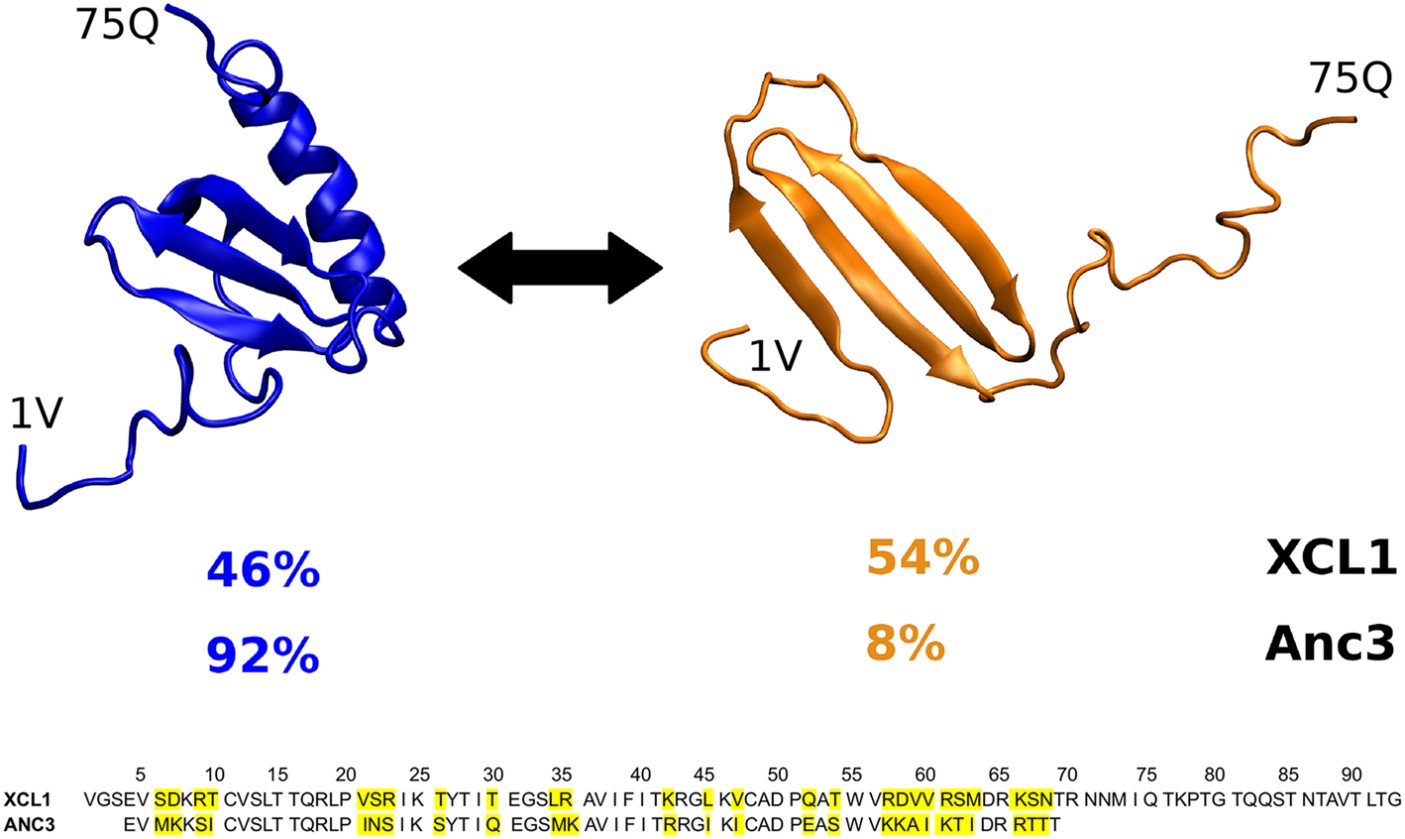
Crystal structures of XCL1-Chemfold (blue, PDB ID 1J8I) and XCL1-Altfold (orange, PDB ID 2JP1). The experimental percentages of the two folded states in XCL1 and in the ancestor (Anc3) are reported

**Fig. 2:**
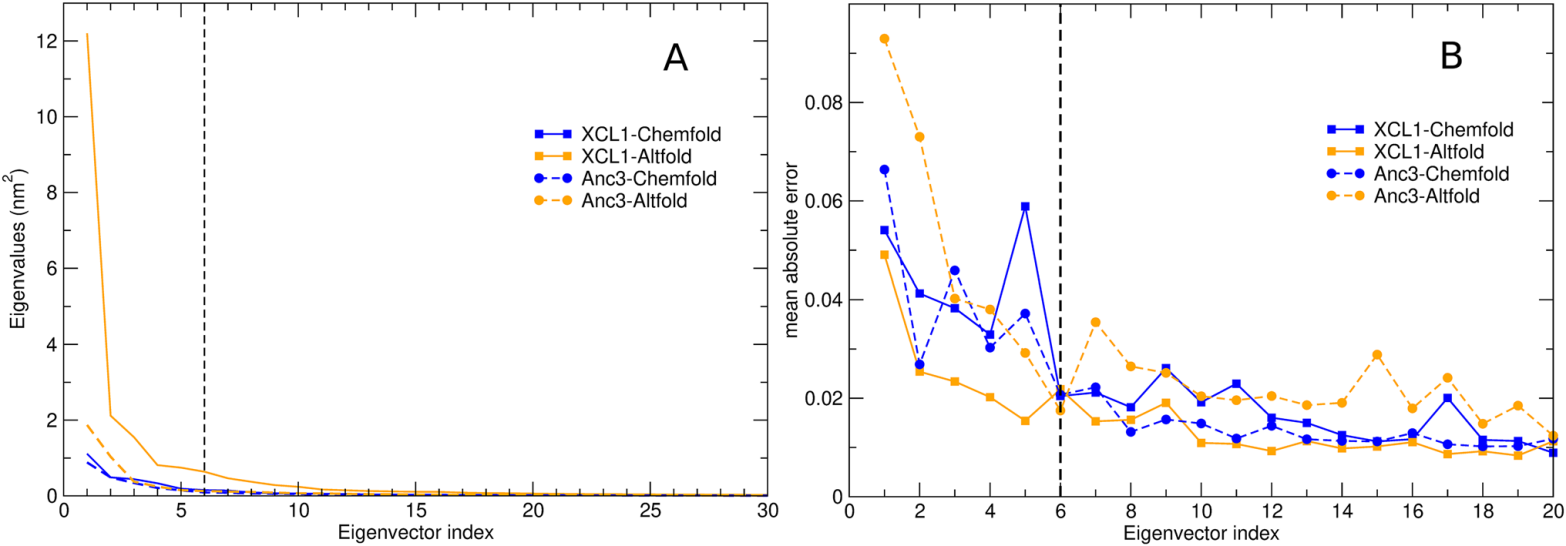
**A** C$_\alpha$ covariance matrix eigenvalues for the Chemfold (blue) and Altfold (orange) conformations of the XCL1 (solid lines) and Anc3 (dashed lines) proteins. **B** Deviation from gaussianity for the distributions of the first 20 eigenvectors obtained from the MD simulations of XCL1 (squares, solid lines) and Anc3 (circles, dashed lines) in the Chemfold (blue) and Altfold (orange) states. The deviation is calculated as the average of the difference between the distribution of the projection of the trajectory on each eigenvector and the corresponding gaussian distribution. The black dashed lines highlights the eigenvector index threshold above which the distribution is assumed gaussian (Color figure online)

**Fig. 3:**
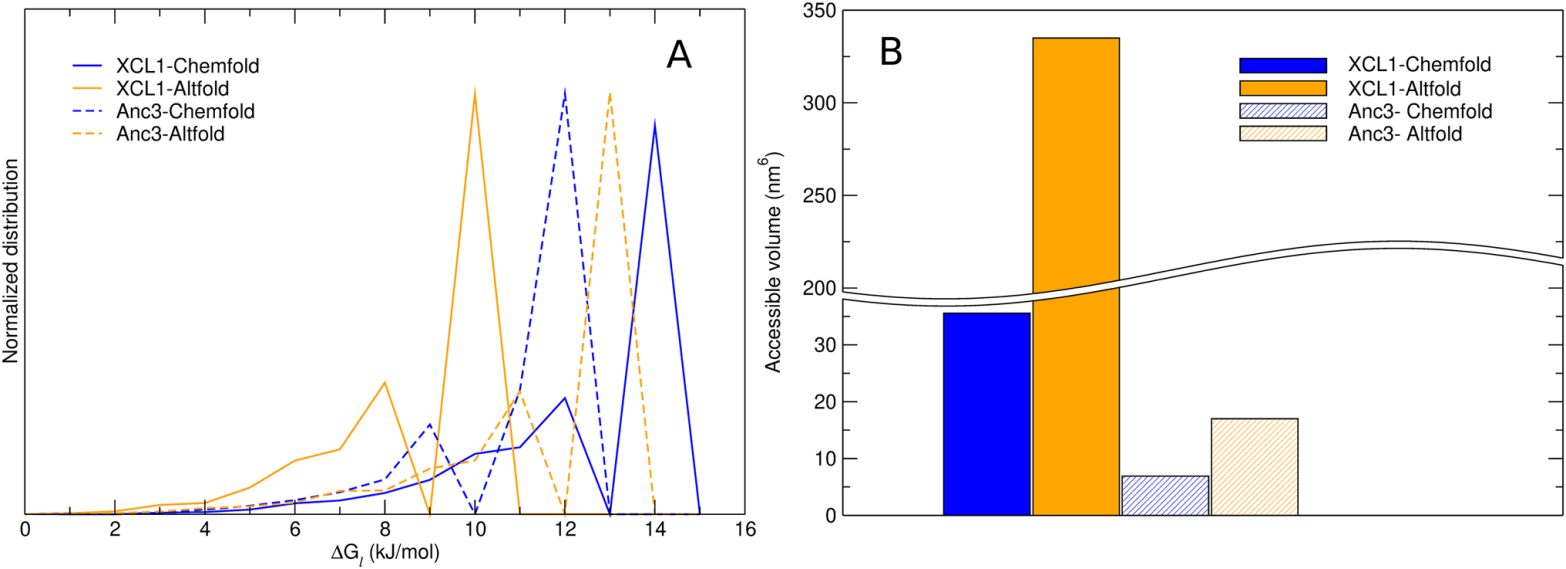
**A** Normalized distribution of the hyper-rectangle free energy (with respect to the minimum) as obtained by projecting the MD trajectories on the hexa-dimensional essential space and evaluating the corresponding probabilities. Blue: Chemfold; orange: Altfold; solid lines: XCL1; dashed lines: Anc3. **B** Accessible volume for the four conformers (XCL1 and Anc3 in both the Chemfold and Altfold states) computed from the numbers of populated hyper-rectangles in the essential space grid (Color figure online)

**Fig. 4:**
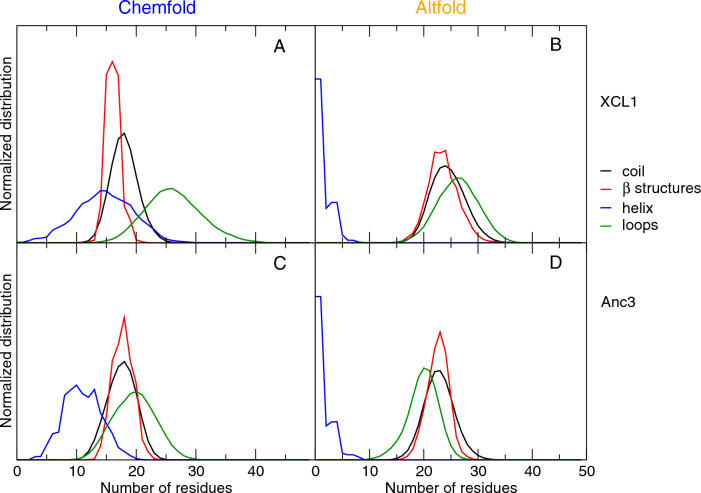
Time evolution of the number of residues with different secondary structure assignements according to the DSSP program [35]. Yellow: unstructured (random coil); red: $\beta$-structures ($\beta$-sheet+$\beta$-bridge); blue: helical structures ($\alpha$-helix+3-10 helix); green: loops (turns+bends). The data are reported for XCL1 (**A** and **B**) and Anc3 (**C** and **D**) for the MD simulations in the Chemfold (**A** and **C**) and Altfold (**B** and **D**) states (Color figure online)

**Fig. 5:**
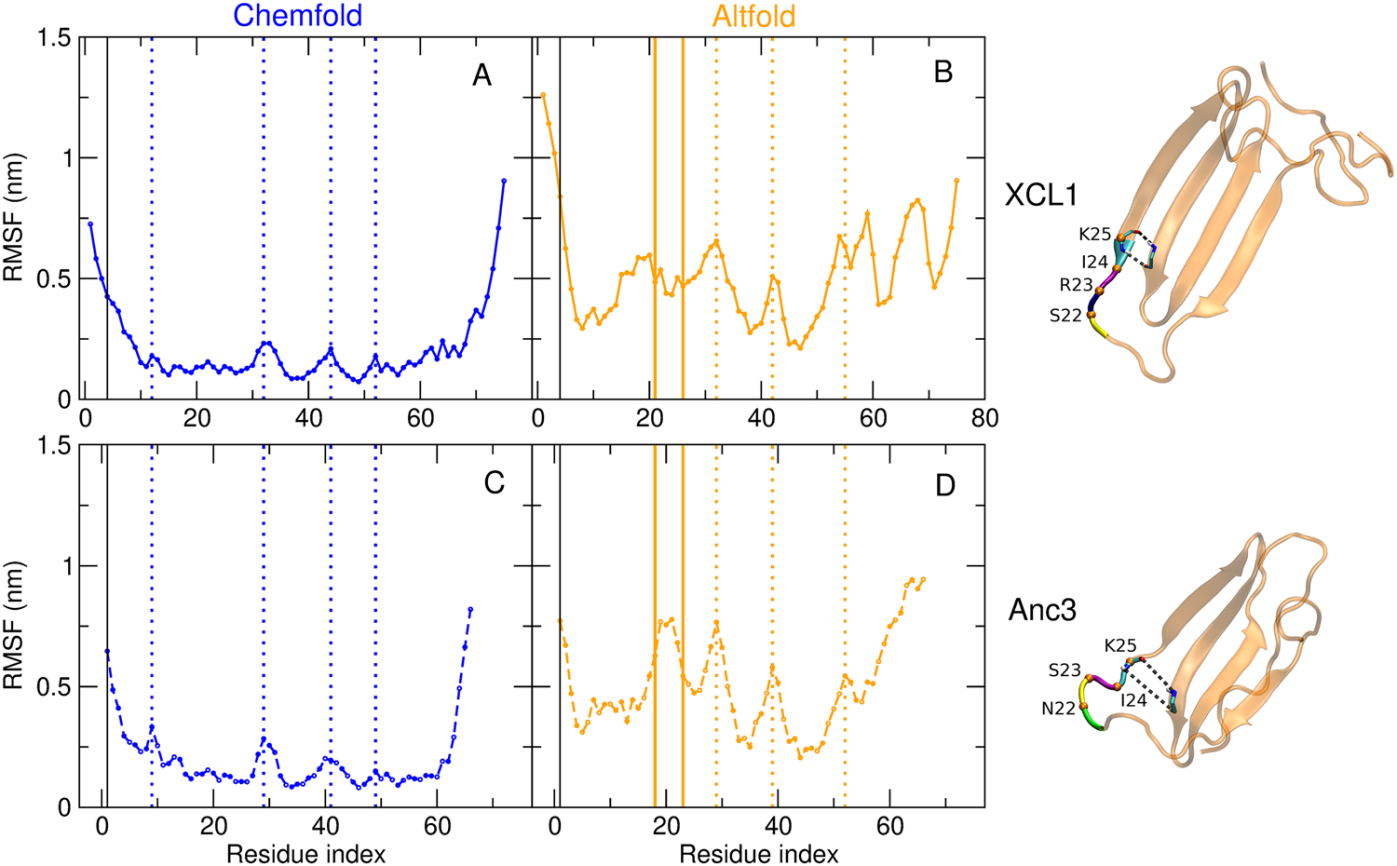
Root mean square fluctuation (RMSF) of the C$_\alpha$ atoms along the MD trajectories of XCL1 and Anc3 in the two folding states. Blue: Chemfold (**A** and **C**); orange: Altfold (**B** and **D**); solid lines: XCL1 (**A** and **B**); dashed lines: Anc3 (**C** and **D**). The vertical black thin solid line marks residue 4 in XCL1 corresponding to residue 1 in Anc3. The orange/blue dotted lines label the residues that show an above average RMSF in both XCL1 and Anc3. The region in which a different RMSF is observed in XCL1- and Anc3-Altfold is highlighted in orange in panels **B** and **D**. Representative snapshots showing the conformational differences in that region are shown next to panels **B** and **D** (Color figure online)

The human chemokine XCL1 (or Lymphotactin), as other metamorphic proteins [8, 9], lies rather in the center of this gradient of protein structural order. XCL1 can reversibly adopt two different, well defined tertiary structures. One comprises three antiparallel $\beta$-sheets packed against the C-terminal $\alpha$-helix, the chemokine fold state (Chemfold), whereas the second consists of a unique fully $\beta$-sheet structure, [10] termed here alternative fold state (Altfold) (see Fig. 1). The Chemfold and the Altfold of XCL1 can modulate immune cells chemotaxis and inflammatory signaling by binding G-protein coupled receptors (GPCR) and glycosaminoglycans (GAGs), respectively. Also, the Altfold can dimerize adding an additional layer of complexity.

**Table 1:** Calculated values of $\Delta \Delta G - \Delta \Delta \mathcal{A}_0 = \Delta G_{XCL1}-\Delta \mathcal{A}_{0,XCL1} - (\Delta G_{Anc3}-\Delta \mathcal{A}_{0,Anc3})$, $\Delta \Delta G_S$ and $\Delta \Delta G_L$ with $\Delta \Delta G = \Delta \Delta \mathcal{A}_0 + \Delta \Delta G_{S}+\Delta \Delta G_{L}$ the conformational free energy change variation for the Altfold $\rightarrow$ Chemfold transition (see the Theory section)

$\Delta \Delta G_{exp}$	$\Delta \Delta G- \Delta \Delta \mathcal{A}_0$	$\Delta \Delta G_{S}$	$\Delta \Delta G_{L}$
6.72	6.80	3.45	3.35

For comparison in the table we also report the experimental value of the conformational free energy change variation $\Delta \Delta G_{exp}$. All values are given in kJ/mol

**Table 2:** Calculated $\Delta G_S$ and $\Delta G_L$ conformational free energy change contributions for XCL1 and Anc3 proteins

	$\Delta G_S$	$\Delta G_L$
XCL1	5.78	3.25
Anc3	2.33	$-$0.10

All values are given in kJ/mol

**Table 3:** Average number of residues, and corresponding standard deviation in parenthesis, identified by the DSSP program as unstructured (coil), belonging to $\beta$ or helical structures and belonging to loops along the MD simulations of XCL1 and Anc3 in both the Chemfold and Altfold states

	Coil	$\beta$-structures	Helix	Loops
XCL1-Chemfold	18.0 (2.1)	16.1 (0.9)	14.7 (4.8)	26.2 (4.6)
Anc3-Chemfold	17.5 (2.3)	17.7 (1.5)	11.1 (3.1)	19.7 (3.4)
XCL1-Altfold	24.4 (3.0)	23.6 (2.7)	0.7 (1.4)	26.3 (3.7)
Anc3-Altfold	22.8 (2.7)	22.7 (1.8)	0.7 (1.5)	19.8 (2.7)

Recently, Dishman et al. investigated the metamorphism of XCL1 from an evolutionary perspective and found that XCL1 became a fold-switching protein $\approx$150 million years ago [11]. Its inferred ancestor displayed only the conventional chemokine fold state (Chemfold). This evolutionary path is consistent with a general trend of decreasing protein structural order observed moving from prokaryotes to eukaryotes [1, 12]. Eventually, the homeostasis development in multicellular organism protected proteins from environmental stress. This decreased the evolutionary pressure for achieving protein structures stable to temperature and pH variations. On the other hand, organisms of increased complexity needed more tight regulation of cellular crosstalk, signaling, and protein expression [1, 2, 12]. This caused an opposite evolutionary pressure and mutation encoding structural disorder became conserved. Increasing protein polymorphism and structural disorder meet the needs of complex organisms without expanding their genome size [1].

Identifying existing metamorphic proteins, like XCL1, and designing novel switch folds is a challenging task from both an experimental and computational perspective [13, 14]. Even AlphaFold 2 (AF2), used to expand the structural coverage of the human proteome beyond experimentally characterized proteins, is inaccurate for disordered and metamorphic proteins [14]. In particular, almost all metamorphic proteins are predicted with a single stable fold by AF2. This algorithm [15] is based on pattern recognition and does not account for protein biophysics. In the case of Lymphotactin, AF2 predicts XCL1 to adopt the conserved $\alpha$+$\beta$ fold of chemokines [16]. In fact, AF2 has been trained on known protein structures including several chemokines. Since AF2 acknowledges some similarity between CXCL1 sequence of those of other known chemokines, it predicts XCL1 to fold like the other monomorphic chemokines. Thus, computational methods capable of providing a link between the conformational thermodynamics of fold switching and the underlying molecular mechanisms [17–19] can help deciphering the molecular bases which define metamorphic proteins.

Previous computational works on human lymphotactin focused on the characterization of the structural conversion between the Chemfold and Altfold in XCL1 [20–23]. It was found that the fold switching does not require unfolding of the protein but rather proceeds through a series of partially-structured intermediates [20] and the contribution of specific electrostatic and hydrophobic interactions to the relative stability of the two folds was addressed [23]. Here, we focus on the thermodynamic evolution of the fold switching in human lymphotactin from an ancestor (in particular Anc3, as defined in [11]) to the current XCL1. Experimentally, it was found that the relative Chemfold/Altfold occupancy in Anc3 is 92/8, while it is almost 50/50 in XCL1. We make use of molecular dynamics (MD) simulations on the microsecond timescale, in conjunction with a thermodynamic model, to obtain the fold switching equilibrium properties in terms of the free energy landscape and the entropic contribution due to the configurational accessible volume.

## Theory and Methods

### Theory

Let us consider a solute-solvent macroscopic system to be used for evaluating the solute thermodynamic properties. When considering only the standard state properties (i.e., disregarding any solute-solute interaction effect) we can conceive, as in the present case, the macroscopic solute-solvent system as defined by only one solute molecule embedded into a huge amount of solvent molecules (i.e., solute infinite dilution). Note that when the activity coefficients are to be considered (i.e., the solute-solute interactions are taken into account) a solute molecule (i.e., the reference solute molecule) must be selected.

Introducing a set of generalized solute internal (classical-like) coordinates, the essential degrees of freedom $\varvec{\xi }$, describing the relevant structural transitions, we can define the corresponding Landau free energy $\mathcal{A}(\varvec{\xi })$ via

1 $$\begin{aligned} \mathcal{A}(\varvec{\xi }) = -k_B T \ln \mathcal{Q}(\varvec{\xi }) \end{aligned}$$

where $\mathcal{Q}(\varvec{\xi })$ is the partition function density providing the system canonical partition function *Q* by

2 $$\begin{aligned} Q = \int \mathcal{Q}(\varvec{\xi }) d \varvec{\xi } = \int e^{-\beta \mathcal{A}(\varvec{\xi })} d \varvec{\xi } \end{aligned}$$

with $1/\beta = k_B T$ ($k_B$ and *T* being the Boltzmann constant and the absolute temperature) and the integral performed over the whole accessible $\varvec{\xi }$ domain of the solute. It is worth noting that for a peptide or a protein a proper choice for the essential coordinates is given by those generalized backbone degrees of freedom providing large and correlated internal motions, as provided by the principal component analysis of the atomic positional fluctuations [24, 25], with thus the Landau free energy basically corresponding to the free energy of the system when the reference solute is fixed at a given $\varvec{\xi }$ position with all the other classical-like coordinates fluctuating according to the equilibrium ensemble. We can dissect the free energy by subdividing the $\varvec{\xi }$ essential space into a large number *N* of hyper-rectangles, each small enough that the corresponding Helmholtz free energy $A_l$ can be well approximated according to Eq. 2 by

3 $$\begin{aligned} A_l= & {} -k_B T \ln Q_l = -k_B T \ln \left\{ \int _{\varvec{\xi }_l} e^{-\beta \mathcal{A}(\varvec{\xi })} d \varvec{\xi } \right\} \nonumber \\\cong & {} -k_B T \ln \{e^{-\beta \mathcal{A}(\varvec{\xi }_l)} \delta \} = \mathcal{A}(\varvec{\xi }_l) -k_B T \ln \delta \end{aligned}$$

with $\delta$ the hyper-rectangle volume used to define the essential space grid and the $\varvec{\xi }_l$ subscript of the integral sign meaning that integration is performed only within the lth grid hyper-rectangle centered at the $\varvec{\xi }_l$ essential space position. Defining with $\mathcal{A}_0 = \mathcal{A}(\varvec{\xi }_0)$ the minimum Landau free energy located at the essential space position $\varvec{\xi }_0$, we can write

4 $$\begin{aligned} Q\cong & {} \sum _{l=0}^{N-1} e^{-\beta \mathcal{A}(\varvec{\xi }_l)} \delta \nonumber \\\cong & {} \delta \; e^{-\beta \mathcal{A}_0} \sum _{l=0}^{N-1} e^{-\beta \Delta A_l} \end{aligned}$$

5 $$\begin{aligned} \Delta A_l= & {} A_l - A_0 \cong \mathcal{A}(\varvec{\xi }_l) - \mathcal{A}_0 \end{aligned}$$

with the summation running over all the accessible grid points and the free energy change $\Delta A_l$ as obtained at fixed system volume.

From Eq. 4 we then readily obtain for any $C1 \rightleftharpoons C2$ conformational equilibrium (in the present case the Altfold and Chemfold conformations) the free energy of each conformation characterized by the corresponding essential space, via

6 $$\begin{aligned} A_{C1}= & {} -k_B T \ln Q_{C1} \cong \mathcal{A}_{C1,0} -k_B T \ln \delta _{C1}\nonumber \\{} & {} \quad -k_B T \ln \left\{ \sum _{l=0}^{N_{C1}-1} e^{-\beta \Delta A_{C1,l}} \right\} \nonumber \\= & {} \mathcal{A}_{C1,0} -k_B T \ln \{ N_{C1}\delta _{C1} \} -k_B T \ln \left\{ \frac{\sum _{l=0}^{N_{C1}-1} e^{-\beta \Delta A_{C1,l}}}{N_{C1}} \right\} \nonumber \\= & {} \mathcal{A}_{C1,0} -k_B T \ln V_{C1} -k_B T \ln \langle e^{-\beta \Delta A_{C1,l}} \rangle _0 \end{aligned}$$

7 $$\begin{aligned} A_{C2}= & {} -k_B T \ln Q_{C2} \cong \mathcal{A}_{C2,0} -k_B T \ln \delta _{C2}\nonumber \\{} & {} \quad -k_B T \ln \left\{ \sum _{l=0}^{N_{C2}-1} e^{-\beta \Delta A_{C2,l}} \right\} \nonumber \\= & {} \mathcal{A}_{C2,0} -k_B T \ln \{ N_{C2}\delta _{C2} \} -k_B T \ln \left\{ \frac{\sum _{l=0}^{N_{C2}-1} e^{-\beta \Delta A_{C2,l}}}{N_{C2}} \right\} \nonumber \\= & {} \mathcal{A}_{C2,0} -k_B T \ln V_{C2} -k_B T \ln \langle e^{-\beta \Delta A_{C2,l}} \rangle _0 \end{aligned}$$

where $V_{C1} = N_{C1} \delta _{C1}, V_{C2}=N_{C2} \delta _{C2}$ are the accessible essential space hyper-volumes of the *C*1 and *C*2 conformation, respectively, and

8 $$\begin{aligned}{} & {} \langle e^{-\beta \Delta A_{C1,l}} \rangle _0 \nonumber \\{} & {} \quad = \frac{1}{N_{C1}} \sum _{l=0}^{N_{C1}-1} e^{-\beta \Delta A_{C1,l}} \end{aligned}$$

9 $$\begin{aligned}{} & {} \langle e^{-\beta \Delta A_{C2,l}} \rangle _0\nonumber \\{} & {} \quad = \frac{1}{N_{C2}} \sum _{l=0}^{N_{C2}-1} e^{-\beta \Delta A_{C2,l}} \end{aligned}$$

with the zero subscript of the angle brackets thus indicating that averaging is performed within the ideal isotropic *C*1 or *C*2 conformation ensemble, as obtained when imposing for each accessible grid point the minimum Landau free energy $\mathcal{A}_{C1,0}$ or $\mathcal{A}_{C2,0}$ (i.e., the free energy lower bound). Note that in the right side of Eqs. 6 and 7 the second term, involving the accessible essential space volume, is purely entropic while the first and third terms (the Landau free energy minimum and the contribution due to the free energy landscape within the essential space) possibly include both entropic and energetic effects. It is also worth noting that from thermodynamics we have $\Delta A_l = \Delta G_l = \Delta \mu _l$ (with the Gibbs free energy change $\Delta G_l$ as obtained at fixed pressure and $\Delta \mu _l$ the corresponding chemical potential change) providing, using Eqs. 6 and 7, the free energy change $A_{C2} - A_{C1} = \Delta A = \Delta G$ for the $C1 \rightarrow C2$ conformational transition

10 $$\begin{aligned} \Delta G= & {} G_{C2} - G_{C1} \cong \Delta \mathcal{A}_0 + \Delta G_S + \Delta G_L \end{aligned}$$

11 $$\begin{aligned} \Delta \mathcal{A}_0= & {} \mathcal{A}_{C2,0} - \mathcal{A}_{C1,0} \end{aligned}$$

12 $$\begin{aligned} \Delta G_S= & {} -k_B T \ln \frac{V_{C2}}{V_{C1}} \end{aligned}$$

13 $$\begin{aligned} \Delta G_L= & {} -k_B T \ln \frac{\langle e^{-\beta \Delta A_{C2,l}} \rangle _0}{\langle e^{-\beta \Delta A_{C1,l}} \rangle _0} \nonumber \\{} & {} \quad = -k_B T \ln \frac{\langle e^{-\beta \Delta G_{C2,l}} \rangle _0}{\langle e^{-\beta \Delta G_{C1,l}} \rangle _0} \end{aligned}$$

14 $$\begin{aligned} \Delta G_{C1,l}= & {} -k_B T \ln (P_{C1,l}/P_{C1,0}) \end{aligned}$$

15 $$\begin{aligned} \Delta G_{C2,l}= & {} -k_B T \ln (P_{C2,l}/P_{C2,0}) \end{aligned}$$

with $P_l$ the equilibrium probability of the *l*th hyper-rectangle as obtained within the isobaric-isothermal ensemble.

### Molecular Dynamics Simulations

MD simulations of both the native protein (XCL1) and the ancestor (Anc3) are performed in both folding states. Four MD simulations are thus performed: XCL1 in the Chemokine fold state (XCL1-Chemfold); XCL1 in the alternative fold state (XCL1-Altfold); Anc3 in the Chemokine fold state (Anc3-Chemfold) and Anc3 in the alternative fold state (Anc3-Altfold). All MD simulations are performed at physiological conditions (310 K, 1 bar and 150 mM NaCl), to match the experimental conditions at which the fraction of Chemokine fold is 0.5 and 0.9 in XCL1 and Anc3, respectively [11].

The starting structure for the XCL1-Chemfold simulation is taken from the NMR structure (PDB ID: 1J8I [26]). In the NMR structure, most of the C-terminal tail is found in an unstructured, extended and highly flexible conformation. Since inclusion of this tail would require a much larger box preventing the proper configurational sampling of the two conformations (thus providing unconverged thermodynamic properties), we do not include the C-terminal disordered tail in our MD simulations. As previously done for the same system [20], we restrict the MD simulation from the 93 residues in the NMR structure to the first 75 residues, i.e. including all the structured fragments in the Chemfold state plus 10 additional unstructured residues.

The initial structure for the XCL1-Altfold is also taken from the NMR structure (PDB ID: 2JP1 [10]). In that structure, only the first 60 residues are resolved. Therefore, we modeled the residues from 61 to 75 in a random-coil configuration. Experimentally, the XCL1-Altfold is found as a dimer in solution. Nonetheless, the formation/rupture of this dimer is a prerequisite for the interconversion between the two folding states and the second process is considered the rate-limiting step [10, 20]. Therefore, we simulate here the monomeric form of XCL1-Altfold.

The starting structures for Anc3-Chemfold and Anc3-Altfold are constructed by homology modeling with the SWISS-MODEL server [27] using as templates the corresponding NMR XCL1 structures (PDB IDs: 1J8I and 2JP1, respectively) and the FASTA sequence of the ancestor [11]. The ancestor sequence comprises only 66 residues (93 in XCL1) with residue 1 in the ancestor corresponding to residue 4 in XCL1. Therefore, to model the Anc3-Altfold starting structure we used the XCL1 as a template for the first 57 residues and modeled the last 9 residues in a random-coil configuration.

All MD simulations are performed using the GROMACS package [28] and the Amber99SB-ILDN force field [29] in the NPT (constant temperature, pressure and number of molecules) ensemble using the velocity rescaling temperature coupling [30] and the Berendsen barostat [31]. The starting configurations are solvated in a dodecahedral TIP3P [32] water box large enough to ensure a minimum distance between the solute atoms and the box edges of $\approx$1 nm. Periodic boundary conditions (PBC) are employed and the long-range electrostatic interactions are treated with the particle mesh Ewald method [33]. A 11 Å  cutoff is used for van der Waals interactions. The LINCS algorithm is used to constrain all covalent bonds involving hydrogens [34]. After a solute optimization and a subsequent solvent relaxation, each system is gradually heated from 50 K to 310 K using short MD simulations (310 K is the temperature used in the experiments to be compared with our data). The length of the four productive runs is 770 ns for XCL1-Chemfold and XCL1-Altfold; 930 ns for Anc3-Chemfold and Anc3-Altfold. For all trajectories, coordinates are saved every 1 ps.

## Results

For both XCL1 and Anc3 we perform extended MD simulations of the two folding states (Chemfold and Altfold) to obtain a proper sampling of the corresponding internal configurational space, and thus to characterize the relevant essential coordinates to be used. We analyze each of the four MD simulations by means of the principal components of the C$_\alpha$ positional fluctuations (Essential Dynamics analysis [19, 24, 25]), obtaining as usual that only a few generalized coordinates provide relevant structural changes (corresponding to the covariance matrix eigenvectors with the largest eigenvalues) with all the other generalized coordinates (corresponding to all the other covariance eigenvectors) characterized by small Gaussian-like and statistically independent fluctuations (i.e., near-constraints) unable to determine relevant structural transitions. It is worth noting that the side chains, just like the solvent, affect the C$_{\alpha }$ dynamical behavior thus being involved in the definition of the C$_{\alpha }$ eigenvectors. The side-chain and solvent effects thus determine the free energy lanscape within the C$_{\alpha }$ essential space as a consequence of the equilibrium side-chain/solvent fluctuation at each essential space position. Moreover, the use of all-atoms eigenvectors instead of the C$_{\alpha }$ ones, although in principle possible and often utilized for peptide conformational analysis, was excluded in our investigation due to the slow convergence of the corresponding all-atoms covariance matrix, preventing its use for evaluating the conformational thermodynamics of proteins. Our analysis shows that for all the cases considered only six covariance eigenvectors (the first six eigenvectors when ordering them according to their decreasing eigenvalues) can provide large and coupled non-Gaussian structural fluctuations, thus defining the essential space to be used, see Fig. 2, panels A and B. Note that the Gaussian near-constraint fluctuations are irrelevant for the variations of the conformational thermodynamic properties within the essential space, as they are independent of the essential space positions. By using for each of the four essential spaces an extended grid covering all the accessible positions, we obtain for both proteins, via Eqs. 10–15, the contributions $\Delta G_S, \Delta G_L$ to the Altfold $\rightarrow$ Chemfold conformational free energy changes ($\Delta G_{XCL1}$ and $\Delta G_{Anc3}$). Only the $\Delta \mathcal{A}_0$ terms could not be evaluated due to the very slow conformational kinetics impeding any sampling of the transitions within the MD simulations and hence not allowing us to estimate $\Delta G_{XCL1}$ and $\Delta G_{Anc3}$. A direct comparison between the calculated and the experimental conformational free energy change is not possible, as in both proteins the Altfold conformation in the experimental conditions is present only in the dimeric form [11], thus providing a conformational free energy change affected by the Altfold dimerization which is neglected in our calculations. However, by considering the difference of the free energy change between XCL1 and Anc3 proteins ($\Delta \Delta G = \Delta G_{XCL1}-\Delta G_{Anc3}$) and assuming similar dimerization free energy contributions, it becomes possible to estimate $\Delta \Delta \mathcal{A}_0 = \Delta \mathcal{A}_{0,XCL1}-\Delta \mathcal{A}_{0,Anc3}$ via

16 $$\begin{aligned}{} & {} \Delta \Delta G_{exp} \approx \Delta \Delta G \nonumber \\{} & {} \quad = \Delta \Delta \mathcal{A}_0 + \Delta \Delta G_S + \Delta \Delta G_L \end{aligned}$$

17 $$\begin{aligned} \Delta \Delta G= & {} \Delta G_{XCL1} - \Delta G_{Anc3} \end{aligned}$$

18 $$\begin{aligned} \Delta \Delta G_S= & {} \Delta G_{S,XCL1} - \Delta G_{S,Anc3} \end{aligned}$$

19 $$\begin{aligned} \Delta \Delta G_L= & {} \Delta G_{L,XCL1} - \Delta G_{L,Anc3} \end{aligned}$$

and hence

20 $$\begin{aligned}{} & {} \Delta \Delta \mathcal{A}_0 \approx \Delta \Delta G_{exp} \nonumber \\{} & {} \quad - \Delta \Delta G_S - \Delta \Delta G_L \end{aligned}$$

with $\Delta \Delta G_{exp}$ the experimental estimate of the free energy change variation between XCL1 and Anc3 proteins, $\Delta \Delta G$ the corresponding value (neglecting the dimerizaton) as obtained by our calculations and $\Delta \Delta G_S, \Delta \Delta G_L$ the calculated free energy terms according to the Theory subsection. From the data reported in Table 1 it follows $\Delta \Delta \mathcal{A}_0 \approx 0$ (see Eq. 20) hence allowing us to disregard the change of the free energy minimum $\Delta \mathcal{A}_0$ when considering the Anc3 $\rightarrow$ XCL1 evolution: i.e., we can consider $\Delta \mathcal{A}_0$, just like the dimerization free energy, as basically the same for XCL1 and Anc3 proteins. It is worth noting that our assumption of similar dimerization effects for the Altfold conformation in both proteins is supported by the fact that among the mutations at the dimer interface, only the I10T mutation (XCL1 numbering) implies a relevant change in hydrophobicity. In addition, it was previously shown that three mutations from polar or charged residues in Anc2 to aliphatic side chains in Anc3 has a limited effect on the stability of the Altfold state [11]. From Table 1 it is evident that the Anc3 $\rightarrow$ XCL1 evolution is characterized by the stabilization of the Altfold conformation compared to the Chemfold one, via both the $\Delta G_S$ and $\Delta G_L$ free energy terms. This is explicitly shown in Table 2 where we report the values of these two free energy contributions for XCL1 and Anc3 proteins. From Fig. 3 it is clear that the increased relative thermodynamic stability of the Altfold conformation in XCL1 is due to the Altfold enlarged accessible essential space volume as well as to the reduced/enhanced free energy variations from the minimum as a function of the essential space position for the Altfold/Chemfold conformations: such modifications of the free energy variation distributions in XCL1 provides an augmented essential space accessibility in the Altfold conformation (i.e., larger partition function) and a decreased essential space accessibility in the Chemfold conformation (i.e., smaller partition function).

Interestingly, from the approximation of virtually identical effects of the Altfold dimerization in XCL1 and Anc3 proteins, resulting in $\Delta \Delta \mathcal{A}_0 \approx 0$, we can express the conformational free energy change for the Altfold $\rightarrow$ Chemfold transition via

21 $$\begin{aligned}{} & {} \Delta G_{XCL1} \approx -\Delta G_{Dim} \nonumber \\{} & {} \quad + \Delta \mathcal{A}_0 + \Delta G_{S,XCL1} + \Delta G_{L,XCL1} \end{aligned}$$

22 $$\begin{aligned}{} & {} \Delta G_{Anc3} \approx -\Delta G_{Dim} \nonumber \\{} & {} \quad + \Delta \mathcal{A}_0 + \Delta G_{S,Anc3} + \Delta G_{L,Anc3} \end{aligned}$$

where $-\Delta G_{Dim} > 0$ is the free energy term due to the Altfold dimerization. From the last equations, defining the experimental conformational free energy change of the proteins via $\Delta G_{exp}^{XCL1}$ and $\Delta G_{exp}^{Anc3}$, we then obtain

23 $$\begin{aligned}{} & {} -\Delta G_{Dim} + \Delta \mathcal{A}_0 \approx \Delta G_{exp}^{XCL1} \nonumber \\{} & {} \quad - \Delta G_{S,XCL1} - \Delta G_{L,XCL1} \nonumber \\{} & {} \quad \approx \Delta G_{exp}^{Anc3} - \Delta G_{S,Anc3} - \Delta G_{L,Anc3} \end{aligned}$$

providing $-\Delta G_{Dim} + \Delta \mathcal{A}_0 \approx -8.6$ kJ/mol.

Remarkably, the enlarged accessible essential space volume in XCL1 (in particular in the Altfold state) does not correspond to a lower stability of the overall secondary structure. As it can be observed from Table 3 and Fig. 4, the average number of residues in $\beta$ or helical structures or in unstructured conformation is essentially the same in the two proteins (note that the higher number of residues belonging to loops in XCL1 is due to the larger number of residues in XCL1, 75, with respect to Anc3, 66). In the Chemfold state it can be even observed a slight increase in the content of helical structure in XCL1 with respect to Anc3. This is also due to the larger number of residues in XCL1: as shown by the DSSP [35] analysis (see Fig. 1 in the Supporting Information, SI), in XCL1 there are a few helical residues in the long C-terminal tail. Nonetheless, it can be observed from Table 3 that in XCL1 the fluctuation of the number of residues in helical (Chemfold) or $\beta$ structures (Altfold) is approximately 1.5 times larger than in Anc3, in accordance with the thermodynamic data showing an enlarged accessible essential space volume.

Preservation of the main structural and dynamical features of each folded state along the Anc3 $\rightarrow$ XCL1 evolution can also be inferred from the root mean square fluctuation (RMSF, see Fig. 5). As a matter of fact, it can be observed that XCL1 and Anc3 feature a similar fluctuation pattern, with the Altfold state showing on average larger fluctuations than the Chemfold state. In addition, in spite of several mutations, the residues showing the largest average fluctuations are the same in both proteins. The only exception is the region between residues 21 and 26 (XCL1 numbering) highlighted in orange in Fig. 5B and D. In this small loop there are 4 mutations (Anc3 $\rightarrow$ XCL1): I21V, N22S, S23R and S26T. In Anc3 residues 22-25 (XCL1 numbering) show a larger fluctuation than those in XCL1. Mutations at sites 22 and 23 are likely at the origin of this increased fluctuation, which in turn determines a weakening in the interactions between K25 and I40 that connect two $\beta$-strands (see representative snapshots in Fig. 5). The probability of the two hydrogen bonds between the backbones of K25 and I40 is in fact $\approx$0.44 in XCL1 and $\approx$0.09 in Anc3. The stronger interaction between these two $\beta$-strands and the decreased fluctuation in the nearby loop might also contribute to the higher stability of the Altfold state in XCL1. It is also worth to remark that a marked difference in the residues associated to the same loop can be observed in the components of the first essential eigenvector in XCL1- and Anc3-Altfold (see Fig. 2 in the SI), suggesting a relevant role of this loop in the overall dynamics of both proteins in the Altfold state.

## Discussion

The results shown and described in the previous section indicate that the Anc3 $\rightarrow$ XCL1 relevant change in the metamorphic equilibrium is largely due to the Altfold conformation dramatic increase of the essential space volume (i.e., over a 10-fold increase, see Fig. 3B), resulting in a drastic entropy increase. Moreover, the Altfold conformation is further stabilized by the relevant change of the free energy landscape within the essential space, providing in XCL1 a flatter free energy surface (see Figure 3A) and thus a lower Altfold conformational free energy (i.e., the corresponding essential space grid points are more accessible, resulting in a larger partition function). It is worth to note that for the Chemfold conformation the Anc3 $\rightarrow$ XCL1 evolution provides somewhat compensating essential space volume and landscape effects, resulting in a less affected conformational free energy. Such Anc3 $\rightarrow$ XCL1 reorganization of the conformational properties provides similar conformational free energy change variations due to both the essential space volume and the free energy landscape (i.e., $\Delta \Delta G_S$ and $\Delta \Delta G_L$, see Table 1). Comparison of our results with the experimental free energy change variation indicates that both the Altfold dimerization free energy $\Delta G_{Dim}$ and the minimum Landau free energy change $\Delta \mathcal{A}_0$ are roughly the same for the Anc3 and XCL1 proteins, with $-\Delta G_{Dim} + \Delta \mathcal{A}_0 \approx -8.6$ kJ/mol. These results suggest that in order to achieve an efficient inter-conversion between the two conformations in the XCL1 protein, nature essentially stabilized the Altfold conformation by increasing its accessible configurational space and flattening the free energy landscape rather than by lowering its minimum Landau free energy, the latter probably prevented by the strict structural requirements of the essential space minimum free energy position. Such enhanced configurational accessibility of the Altfold conformation (essentially providing an entropic increase) furnished the compensation for the excess thermodynamic stability of the Chemfold conformation in the Anc3 protein, mainly due to $\Delta \mathcal{A}_0$ (note that being $-\Delta G_{Dim} > 0$ we necessarily have $\Delta \mathcal{A}_0 < -8.6$ kJ/mol).

## Supplementary Information

Below is the link to the electronic supplementary material.

Secondary structure of XCL1 and Anc3 in the Chemfold state and the first eigenvector components of XCL1 and Anc3 in the Altfold state are provided in the Supporting Information (PDF 1765 kb)
