# Supplementary material for: Thermodynamic Evolution of a Metamorphic Protein: A Theoretical-Computational Study of Human Lymphotactin
Source: Protein J. 2023 May 26;42(3):219–28. doi: 10.1007/s10930-023-10123-7 (PMC10264484; doi:10.1007/s10930-023-10123-7)
Supplement: Supplementary file 1 — Secondary structure of XCL1 and Anc3 in the Chemfold state and the first eigenvector components of XCL1 and Anc3 in the Altfold state are provided in the Supporting Information (PDF 1765 kb) [file 10930_2023_10123_MOESM1_ESM.pdf]

Supporting Information for: Thermodynamic evolution of a metamorphic protein: A theoretical-computational study of human Lymphotactin

Laura Zanetti-Polzi[1], Isabella Daidone[2], Claudio Iacobucci[2] and Andrea Amadei[3*]

[1]Center S3, CNR-Institute of Nanoscience, Via Campi 213/A, Modena, 100190, Italy.
[2]Department of Physical and Chemical Sciences, University of L'Aquila, via Vetoio (Coppito 1), L'Aquila, 67010, Italy.
[3*]Department of Chemical and Technological Sciences, University of Rome "Tor Vergata", Via della Ricerca Scientifica 1, Rome, 00185, Italy.

*Corresponding author(s). E-mail(s): andrea.amadei@uniroma2.it;

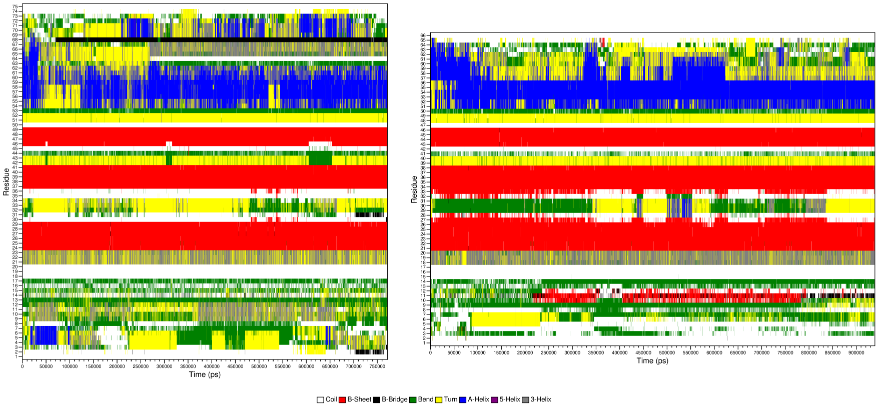

Fig. 1 Secondary structure of XCL1 (left) and Anc3 (right) in the Chemfold state as provided by the DSSP [1] analysis

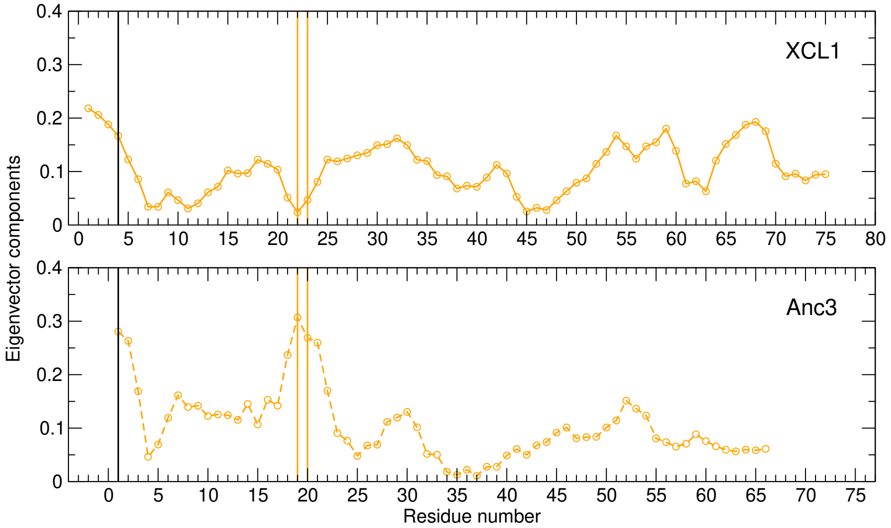

Fig. 2 Per-residue component (i.e., the norm of the vector defined by the x, y, z eigenvector components of each C_α) of the first eigenvector for XCL1 (top) and Anc3 (bottom). The vertical black line marks residue 4 in XCL1 corresponding to residue 1 in Anc3. The orange vertical lines label residues 22 and 23 (XCL1 numbering).

References

[1] Kabsch, W., Sander, C.: Dictionary of protein secondary structure: pattern recognition of hydrogen-bonded and geometrical features. Biopolymers: Original Research on Biomolecules **22**(12), 2577–2637 (1983)
